# Who presents past the gestational age limit for first trimester abortion in the public sector in Mexico City?

**DOI:** 10.1371/journal.pone.0192547

**Published:** 2018-02-07

**Authors:** Biani Saavedra-Avendano, Raffaela Schiavon, Patricio Sanhueza, Ranulfo Rios-Polanco, Laura Garcia-Martinez, Blair G. Darney

**Affiliations:** 1 National Institute of Public Health (INSP), Cuernavaca, Morelos, Mexico; 2 International Pregnancy Advisory Services (Ipas-Mexico), Mexico City, Mexico; 3 Secretaría de Salud de la Ciudad de México, Mexico City, Mexico; University of North Carolina at Chapel Hill, UNITED STATES

## Abstract

**Objective:**

To identify socio-demographic factors associated with presenting for abortion services past the gestational age (GA) limit (12 weeks), and thus not receiving services, in Mexico City’s public sector first trimester abortion program.

**Methods:**

We used clinical data from four high volume sites in the Interrupción Legal de Embarazo (ILE) program, 2007–2015. We used descriptive statistics to quantify the proportion of women who did not receive an abortion due to presenting past the gestational age limit. We used multivariable logistic regression to identify associations between women’s characteristics and presenting past the GA limit and calculated predicted probabilities of late presentation for key characteristics.

**Results:**

Our sample included 52,391 women, 8.10% (n = 4,246) of whom did not receive abortion services due to presenting past the GA limit. Adolescents (12–17) made up 8.69% of the total sample and 13.40% of those presenting past the GA limit (p< 0.05). In multivariable analyses, all age groups of adult women had significantly lower odds than adolescents of presenting past the limit (aOR = 0.77, aOR = 0.63, aOR = 0.58 and aOR = 0.37 for 19–24, 25–29, 30–39, and > = 40 years’ old respectively). Women living in Mexico City and with higher levels of education had lower odds of presenting past the GA limit, and there was an educational gradient across all age groups. In the multivariable predicted probability models, adolescents at every level of education have significantly higher probabilities of not receiving an abortion due to presenting past the gestational age limit compared with adults (among women with a primary education: 11.75% adolescents vs. 9.02–4.26% across adult age groups).

**Conclusions:**

Our results suggest that continued efforts are needed to educate women, especially younger and less educated women, about early pregnancy recognition. In addition, all women need information about the availability of first trimester legal abortion to ensure timely access to abortion services.

## Introduction

Access to legal and safe abortion services is crucial to reducing abortion-related morbidity and mortality and expanding human rights [1]. In Mexico, abortion law is determined at the state level; first trimester abortion was decriminalized in Mexico City, one of Mexico’s 32 states, in 2007. First trimester abortion remains highly restricted in Mexico’s other 31 states. In Mexico City, legal abortion services are available in public and private sectors. The public sector abortion program, called “Interrupcion Legal de Embarazo” (ILE) [2], has provided services for over 188,000 women since program inception in 2007 [3]. In order to receive abortion services women must present official identification, and adolescents (under 18 years old) must be accompanied by a parent or guardian. Women who present for care past 12 weeks’ gestational age (GA) are not eligible to receive services in the ILE program, and second trimester abortion is available only under narrow exceptions requiring documentation (rape, danger to the woman's life and congenital malformations) [4].

Despite the decriminalization of first trimester abortion in Mexico City and the provision of free (for Mexico City residents) or low cost (sliding-scale for women living in other states) services, women continue to face obstacles when attempting to access legal first trimester abortion services. A previous study with a small sample of women who received abortions in the ILE program reported that unmarried women, those living outside Mexico City, and women with lower educational level had trouble accessing services, although they ultimately did [5]. There is no information about the women who are not able to access services—who did not receive a wanted abortion.

Previous research in the United States (US), United Kingdom (UK) and Colombia suggests that adolescent age, unemployment, nulliparity, poverty, low educational level, and living further from the clinic are associated with having second trimester abortions [6–9]. Reasons for delay include difficulties in pregnancy recognition as well as financial and logistical constraints [7–12].

There is little evidence about the proportion of women who present past the GA limit (and thus are not eligible to receive abortion services) in low and middle income countries where abortion has been decriminalized. The purpose of this study was to quantify the proportion of women presenting for ILE services past the GA limit and identify factors associated with presenting past the GA limit and thus not receiving services. Based on previous literature, we hypothesized that younger age and lower education are associated with presenting for abortion services past the GA limit in the Mexico City public abortion program.

## Methods

We conducted a retrospective cohort study using clinical data from four public facilities in the Mexico City ILE program: two primary care clinics and two hospital-based clinics. We generated a database pooling information from two sources: a) an existing electronic database containing information from paper medical records of women who requested abortion services from 2008 to 2012 in two ILE clinics (n = 43,139); and b) a database created by our research team with information extracted from paper medical records between 2007 and 2015 in two hospitals with high ILE volume (n = 11,938). Hospital chart abstractors were trained by the study team how and where to identify relevant information within the chart. The study team developed a web-based platform to capture the data and data were downloaded daily. We performed a 5.00% random re-abstraction of charts and data quality validation exercise to assess the quality of the data abstracted from paper charts, which included all records from the hospital- based clinics (n = 11,938). Overall inter-rater reliability was high, with an average concordance of 95.51% and an average kappa value of 0.93 (S1 Table).

Our final dataset includes clinical data (ultrasound, type of abortion -medication or aspiration) and socio-demographic information on every woman who presented requesting abortion services. We used ultrasound data for GA, or the self-report of last menstrual period when ultrasound information was missing (1.50% of charts, n = 838). However, 1.10% (n = 560) of the sample did not have any gestational age information (neither ultrasound nor last menstrual period). These charts did include a checkbox for information on procedure received or whether the woman did not receive an abortion due to presenting past the GA limit (60.01% or 338/560 without GA data). So, although we do not know exact GA for these observations, we do know they did not receive abortions due to presenting past the GA limit. We therefore chose to retain these observations in our analysis. We excluded women who were eligible to receive abortion services in the ILE program based on GA, but did not end up having an abortion. Most of these were for unknown reasons (1.95%; n = 1,077), but some charts included specific reasons: referred to other institution (0.13%: n = 71), suspected ectopic pregnancy (0.99% n = 544), or were found to not be pregnant after Beta HCG testing (1.80%; n = 994). After these exclusions, our final analytical sample is 52,391 observations.

In order to identify possible biases in our sample, we compared our descriptive results with ILE’s program official statistics 2007–2016 [3], which provide limited aggregate socio-demographic characteristics of all users (N = 187,833). Our sample was quite similar; differences between our sample and all ILE users in all cases were less than 1.5 percentage points. We only found important differences between our sample and aggregate official data in the percentage of adolescents (7.30% in our sample vs. 4.90% overall); however, our sample included a site that serves as a referral site for adolescents and we therefore anticipated adolescents would be overrepresented in our sample compared to all ILE users.

Our primary outcome was not receiving an abortion due to presenting past the 12 week GA limit, versus receiving an abortion. We included several independent variables. We grouped age as 12–17, 18–24, 25–29, 30–39, and 40 or more years. We grouped age this way because women under 18 must have parental or legal guardian consent to receive services, and it was thus important to be able to identify this group separately. We included additional socio-demographic characteristics: marital status (never married, married/cohabited, and divorced/widowed); educational level (completed primary school or less, secondary/9^th^ grade, high school/12^th^ grade, and greater than high school); occupation (whether the woman reported any type of employment outside the home or being a student); number of pregnancies (one/index, 2–3, 4 or more); and state of residence (Mexico City, State of Mexico, and the 30 states). Although we have data spanning 2007–2015, the majority of the data (70.90%) were from 2010–2012, so we controlled for year as a linear variable.

We used descriptive statistics and bivariate tests (χ^2^ test) to examine differences in socio-demographic characteristics of women who did not receive an abortion due to presenting past the GA limit with those who received the abortion they sought. We used multivariable logistic regression to identify associations between women’s characteristics and our primary outcome. We clustered our data at the health facility level to account for non-independence of observations within the same facility [13]. We calculated predicted probabilities [14] of presenting past the gestational age limit for key combinations of variables (education and age and number of pregnancies), holding the covariates included in the multivariable regression model constant, to ease interpretation.

We performed several sensitivity analyses. We did not have complete socio-demographic data on a substantial proportion of women who did not receive services (see Table 1)–although each woman had a chart opened, charts were not routinely completed once it was determined that the woman was over the GA limit. We therefore examined models with and without the covariates with missing data; results were unchanged, S2 Table shows differences between observations included in our model and those with missing data (N = 3,180/ 52,399; 6.06%).

**Table 1:** Socio-demographic characteristics of women who sought legal abortion services at ILE program.

	Did not receive an abortion due to presenting past the GA limit	Had an abortion	Total
	8.10 (n = 4,246)	91.90 (n = 48,145)	100.00 (n = 52,391)
	%		
Age**			
12–17	13.40	8.28	8.69
18–24	49.93	47.31	47.52
25–29	18.70	21.47	21.24
30–39	15.87	20.09	19.75
> = 40	1.65	2.71	2.63
*missing*	0.45	0.14	0.17
**Marital Status**			
Never Married	34.93	42.48	41.87
Married/ cohabited	41.40	50.57	49.83
Divorced / widowed	4.64	5.35	5.29
*missing*	19.03	1.60	3.01
**Educational level** **			
Primary or lower	8.71	8.66	8.66
Secondary/ 9th grade	31.51	32.95	32.84
High school/ 12th grade	32.67	38.76	38.27
Greater than high school	8.15	17.28	16.54
*missing*	18.96	2.34	3.69
**Occupation** **			
Unemployed, housewife	19.62	24.51	24.12
Employed	38.25	46.39	45.73
Student	22.68	27.17	26.80
*missing*	19.45	1.93	3.35
**State of residence** *			
Mexico City	68.65	71.10	70.90
State of Mexico	25.22	23.87	23.90
Other State	6.12	5.03	5.12
**Number of pregnancies** **			
1	32.64	37.23	36.86
2–3	33.04	44.52	43.59
> = 4	12.08	17.50	17.06
*missing*	22.23	0.74	2.49
Chart Year**			
2007–2009	28.97	20.44	21.14
2010–2012	66.11	71.90	71.43
2013–2015	4.90	7.62	7.40
*missing*	0.02	0.04	0.03

Note:

** p<0.01,

* p<0.05

We tested a model excluding the observations with missing values for GA (n = 560, described above), and results did not change. We control models for year, results did not change when we introduced year as categorical or as continuous variable, we selected the model with year as continuous to ease interpretation. We ran a model with a binary state of residence variable (Mexico City and the other 31 states). We compared all models using the Bayesian Information Criterion (BIC); a smaller value indicates a better fit among a discrete set of models [15]. We selected the complete case model with residence in three categories based on the BIC values. We added a deprivation index—a composite measure used by the Mexican government [16] that includes education, household materials, living environment and poverty—at the municipality level. However, because most of our sample resides in Mexico City (an urban setting with large disparities but low average deprivation) we did not have enough variability to render this variable useful, so we discarded it. We tested models stratified by health facility and results were unchanged. All models were robust to these changes and we present only our final model. This study was approved by the Research Ethics Committee at the National Institute of Public Health (1746), Cuernavaca, the Research and Teaching Committee at Secretaria de Salud (101-110-12-15), Mexico City, and the Oregon Health & Science University IRB. IRB waived the need for participants consent, data was accessed anonymously.

## Results

Our final analytical sample included 52,391 women. Overall, 8.10% (n = 4,246) did not receive abortion services due to presenting past the gestational age limit. Of the 91.90% women who received the abortion they sought, 77.70% had a medication and 22.30% an aspiration abortion. Adolescents (12–17) made up 8.69% of the overall sample. Just over 41.87% of women were never married, 29.02% resided outside of Mexico City (23.90% in state of Mexico and 5.12% were from other states), and 36.86% had not experienced a pregnancy previous to the one they were seeking to terminate (Table 1).

The group of women who did not receive an abortion due to presenting past the GA limit have a greater proportion of adolescent women compared with women who received abortion services (13.40% versus 8.28%; p<0.01; Table 1); a lower proportion had greater than high school education (8.15% vs. 17.28%; p<0.01) compared to those who received abortion services (Table 1). Women who did not receive an abortion were slightly less likely to reside in Mexico City (68.65% vs. 71.10% vs. who receive an abortion; p<0.05).

**Fig 1:**
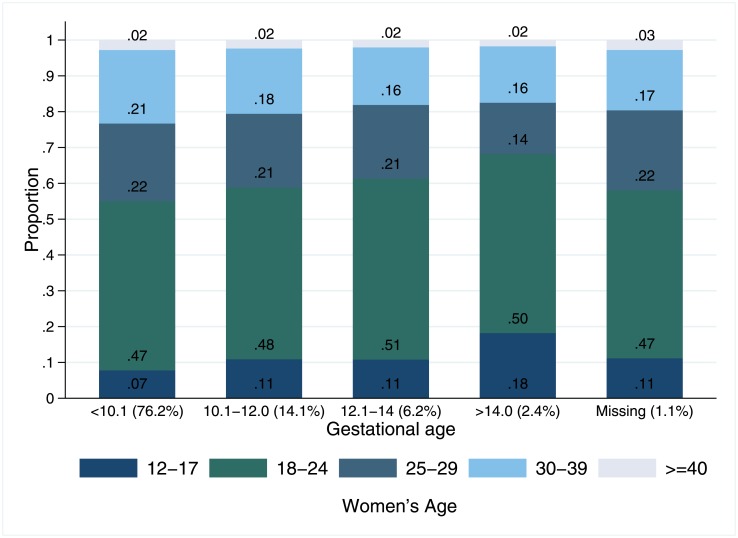
Distribution of age of the women seeking legal abortion services al ILE program, by gestational age. Note: Proportion of the sample in each gestational age group in parenthesis, Chi square test by age group *p<0.05. 1.10% (n = 560) of the sample did not have gestational age information, 60.01% did not receive an abortion due to presenting past the GA limit.

Overall, most women sought abortion services earlier than 10.1 weeks (76.20%); 14.08% between 10.1 and 12.0 weeks; 6.22% between 12.10 and 14.00 weeks of gestation, 2.40% women requested abortion services after 14 weeks of gestation, and 1.10% did not have GA information (Fig 1). Among those women who sought abortion services past 14 weeks of gestation, 18.00% were aged 12 to 17 years; adolescents were thus overrepresented in this group (p<0.05; Fig 1).

In the multivariable model (complete cases n = 49,211) compared with adolescents, all age groups of adult women had lower odds of not receiving an abortion due to presenting for abortion services past the gestational limit, holding other socio-demographic characteristics at the mean. We found that the older the women, the greater the negative association compared with adolescents (aOR = 0.77 for women aged 19–24, aOR = 0.63 for those aged 25–29, aOR = 0.58 for women 30–39, and aOR = 0.37 for women > = 40 years old; Table 2). We identified the same pattern with education level (Table 2). Women who resided in the State of Mexico or other state had higher odds of not receiving an abortion due to presenting past the limit compared with women living in Mexico City (aOR = 1.09; 95% CI = 1.06–1.13; aOR = 1.27; 95% CI = 0.91–1.76 respectively), controlling for covariates.

**Table 2:** Multivariable analysis of associations between presenting for legal abortion services past the gestational limit at ILE program and socio-demographic characteristics, n = 49,211.

	OR	CI 95%
**Age (REF = 12–17)**		
18–24	0.77**	[0.648–0.906]
25–29	0.63**	[0.466–0.857]
30–39	0.58**	[0.425–0.783]
> = 40	0.37*	[0.153–0.885]
**Marital status (REF = Never married)**		
Married/ cohabited	0.96	[0.773–1.182]
Divorced / widowed	1.19	[0.989–1.432]
**Educational level (REF = primary or lower)**		
Secondary/ 9th grade	0.85**	[0.757–0.950]
High school/ 12th grade	0.67**	[0.583–0.781]
Greater than high school	0.40**	[0.343–0.455]
**Occupation (REF = Unemployed, housewife)**		
Employed	0.95	[0.565–1.590]
Student	0.89	[0.549–1.433]
**Number of pregnancies (RFE = 1)**		
2–3	0.86	[0.613–1.196]
> = 4	0.8	[0.554–1.163]
**State of residence (REF = Mexico City)**		
State of Mexico	1.09**	[1.056–1.134]
Other state	1.27	[0.917–1.769]
**Chart Year**	1.02	[0.926–1.120]

Note:

** p<0.01,

* p<0.05

**Fig 2:**
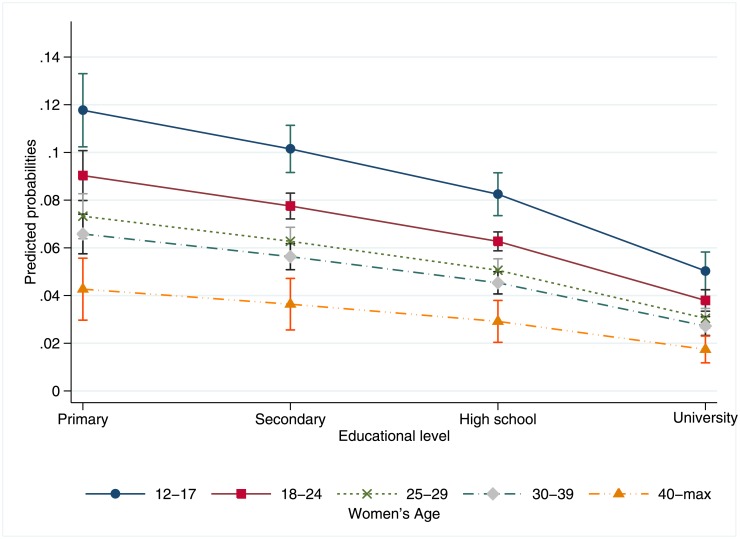
Adjusted predicted probabilities of presenting for legal abortion services al ILE program past the gestational limit by age and educational level, total sample n = 49,211. Note: 95% Confidence Interval.

Among each age group, the relationship of education and not receiving an abortion due to presenting past the gestational limit held. In the multivariable predicted probability models, adolescents with a primary level education had a 11.75% (95% CI = 10.22–13.28%) probability of presenting past the limit, holding other covariates presenting in the multivariable regression model at the mean (Fig 2). Adolescents with a secondary education had a 10.15% (95% CI = 9.16–11.13%) probability, and those with a high school education had an 8.24% (95% CI = 7.34–9.14%) probability of not receiving an abortion due to presenting past the GA; this last group equals the overall crude proportion of the sample who did not receive an abortion due to presenting past the limit. Adolescents at every level of education have significantly higher probabilities of not receiving an abortion due to presenting past the GA limit, controlling for covariates. This general gradient relationship held for all age groups (Fig 2).

**Fig 3:**
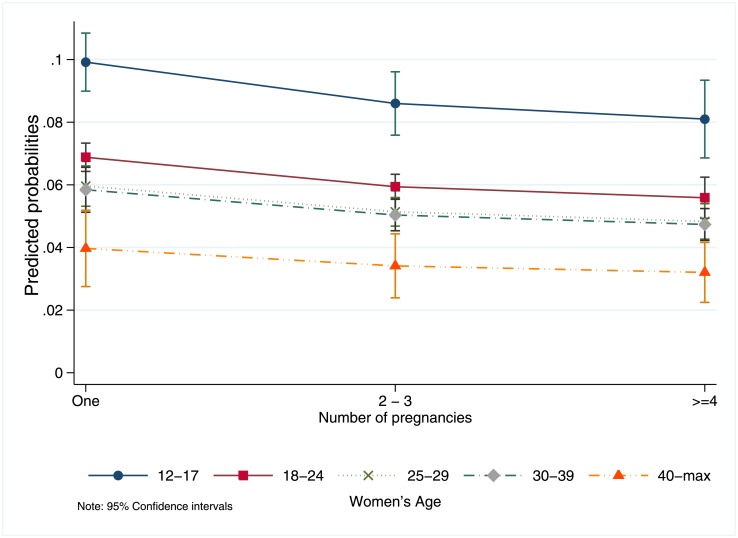
Adjusted predicted probabilities of presenting for legal abortion services al ILE program past the gestational limit, by age and number of pregnancies, total sample n = 49,211. Note: 95% Confidence Interval.

Adolescents also had consistently higher probability of not receiving an abortion due to presenting past the gestational age limit regardless of previous parity (Fig 3). While adolescents with no previous pregnancies had the highest probability (0.99%; 95% CI = 8.98–10.83%) of not receiving an abortion due to presenting past the GA limit among all age groups, controlling for covariates, even adolescents with more than two previous pregnancies had a higher probability of presenting late (8.61%; 95% CI = 7.60–9.62%) compared with older women with no previous pregnancies (range 3.95–6.87%; Fig 3).

## Discussion

We report important disparities in who presents past the gestational age limit, and is thus not able to receive services, in the public legal abortion program in Mexico City (ILE). These disparities in late presentation translate directly into disparities in obtaining wanted abortion services. Adolescent age, lower educational level, and residing outside of Mexico City were associated with presenting for ILE services past 12 weeks’ GA. Local evidence from low and middle income countries about which women seek care past the GA limit can be used for education and advocacy to develop target interventions to both ensure that all women of reproductive age are aware of the availability of legal first trimester abortion and the GA limit, and facilitate early entry into care, especially for adolescent women.

Our results are consistent with previous studies in Colombia and the US focused on factors associated with seeking second trimester abortion, and which identified adolescent age, low educational level, and living further from the clinic as factors in seeking care in the second trimester [6–8]. Delayed awareness of pregnancy has also been found to be a key factor in delay presenting for services [7–12]. Adolescents have been shown to take longer than adult women to suspect and confirm their pregnancy [11, 17], possibly due to irregular menstrual cycle, atypical menstrual bleeding, and not presenting or recognizing pregnancy symptoms [8, 9, 18]. Adolescent age, unwanted or mistimed pregnancy, lower educational level, and poverty were associated with later pregnancy awareness (at 7 weeks’ gestation or later) in a US sample, and the disparity in later pregnancy recognition between adolescents and adult women has not changed over time [17]. In Mexico, disparities in access to and quality of prenatal care between adolescents and adult women have been documented [19, 20], with adolescent women consistently presenting later for care. This context, in conjunction with our finding, suggests that one factor in presenting past the gestational age limit for ILE services is delayed recognition of pregnancy among adolescents in Mexico.

Economic and logistical constraints, lack of information about providers, [7, 8, 10, 11, 21], and geographical characteristics [8, 10] have been identified as key barriers to seeking first trimester and presenting for second trimester abortion in the US and Colombia. We also identified distance (residing outside Mexico City) as a barrier. Women living outside Mexico City likely face geographic, economic and logistical barriers to seeking abortion services. Although we did not find significant disparities between residents of Mexico City and those from the other 30 states (the association was significant only for women living in State of Mexico), our sample was small and we hypothesize that the most vulnerable women—young, poor, less educated, exactly those more likely to present past the GA limit—living outside Mexico City and State are not able to travel to seek ILE program abortion services. Previous studies have also documented distance as a factor in the decision to seek health care in the first place, as well as delay for abortion [22, 23] and maternity [24] care. The effect of distance becomes stronger when combined with poverty or other lack of resources [22–24].

Consistently, studies have identified “deciding to have an abortion” as the shorter delay, relative to other factors [7–10, 21]. It is not the decision process but pregnancy recognition and the logistics of seeking abortion that constitute barriers to seeking timely first trimester care. In contexts where adolescents must have parental or legal guardian consent and accompaniment in order to receive abortion services, as in Mexico City, the decision-making process could be harder due to reluctance to disclose to or opposition from family members [18].

Finally, lack of information about abortion laws and public abortion services in Mexico City and abortion-related stigma might be also important barriers [25, 26]. Women’s knowledge about Mexico City’s abortion legislation is poorly documented, however a study published in 2002, prior to the decriminalization of first trimester abortion in Mexico City, based on a nationally-representative sample of men and women aged 15 to 24 found that 54.00% of participants did not know the legal status of abortion in their state [27]. Our clinical data unfortunately did not allow us to assess abortion legislation knowledge in our study.

While previous literature has focused on reasons for delay seeking abortion, or compared characteristics first and second trimester abortion patients [7], little evidence exists about the proportion of women denied wanted abortions due presenting past the legal GA limit in low and middle income countries. Data from other countries [28–31], primarily qualitative, focuses on reasons for being denied and whether women were able to ultimately interrupt their pregnancy. Our study is the first to estimate the overall proportion that present late for legal first trimester public abortion services.

Our study shares limitations common to all retrospective observational studies. Our data do not permit us to understand why the women who present past the limit do so; we can only identify associations. We do not have nuanced socio-economic data, but educational level is a good proxy for wealth in Mexico [32]. While our dataset is large and represents abortion services in both hospital and clinic settings, it is not exhaustive and does not represent all abortions in the ILE program. The ILE data are fragmented; no single comprehensive patient-level dataset exists. In this study, we made an effort to compile a comprehensive and representative dataset.

The ILE program in Mexico City, which provides legal first trimester abortion free of charge, is a key health system achievement to advance the health and rights of women in Mexico. However, disparities persist in who is able to access services. First trimester abortion is a time-sensitive health service and women need information and skills to recognize pregnancy and access services in a timely manner. This is especially crucial where access to second trimester abortion is restricted to few indications or illegal, as it is in Mexico City, the 31 other states of Mexico, and most low and middle income countries. Education and advocacy efforts can focus on early recognition of pregnancy as one strategy to help women reap the full benefits of legal and free first trimester abortion in Mexico City, and target adolescents, who are most at risk for presenting past the gestational age limit. Our findings can inform efforts in Mexico as well as in other countries to provide the widest possible access to legal first trimester abortion services.

## Supporting information

S1 Table Chart abstraction data validation. (DOCX)

S2 Table Socio-demographic characteristics of women included in the logistic regression model and those women dropped due to missing data. Note: ** p<0.01, * p<0.05 for difference between those included and those dropped.
(DOCX)
