# Supplementary material for: Who presents past the gestational age limit for first trimester abortion in the public sector in Mexico City?
Source: PLoS One. 2018 Feb 7;13(2):e0192547. doi: 10.1371/journal.pone.0192547 (PMC5802931; doi:10.1371/journal.pone.0192547)
Supplement: S1 Table — (DOCX) [file pone.0192547.s001.docx]

**Who presents past the gestational age limit for first trimester abortion in the public sector in Mexico City?**

***Supporting information***

**S1 Table. Chart abstraction data validation**

| **Variable** | **Kappa Statistic** | **Concordance** |
| --- | --- | --- |
| Age | 1.000 | 1.000 |
| Site | 1.000 | 1.000 |
| Year | 1.000 | 1.000 |
| Relationship | 0.980 | 0.987 |
| State | 0.948 | 0.982 |
| Number of Births | 0.965 | 0.979 |
| Occupation | 0.959 | 0.970 |
| Municipality | 0.967 | 0.970 |
| Weeks Pregnant (Menstruation) | 0.962 | 0.967 |
| Cesarean Sections | 0.928 | 0.966 |
| Number of Pregnancies | 0.939 | 0.957 |
| Days Pregnant (Menstruation) | 0.941 | 0.950 |
| Postal Code | 0.947 | 0.947 |
| Abortions | 0.822 | 0.943 |
| ILE Outcome | 0.839 | 0.940 |
| Education | 0.903 | 0.934 |
| Weeks Pregnant (Ultrasound) | 0.907 | 0.920 |
| Days Pregnant (Ultrasound) | 0.887 | 0.904 |
| Smoking | 0.899 | 0.899 |
| Contraceptives | 0.850 | 0.880 |
| **Average** | **0.932** | **0.955** |
