# Supplementary material for: Who presents past the gestational age limit for first trimester abortion in the public sector in Mexico City?
Source: PLoS One. 2018 Feb 7;13(2):e0192547. doi: 10.1371/journal.pone.0192547 (PMC5802931; doi:10.1371/journal.pone.0192547)
Supplement: S2 Table — Note: ** p<0.01, * p<0.05 for difference between those included and those dropped. (DOCX) [file pone.0192547.s002.docx]

**Who presents past the gestational age limit for first trimester abortion in the public sector in Mexico City?**

***Supporting information***

**S2 Table. Socio-demographic characteristics of women included in the logistic regression model and those women dropped due to missing data.**

|  | **In the model** | | | **Dropped** |
| --- | --- | --- | --- | --- |
|  | | 93.04% (n=49,211) | | 6.06% (n=3,180) |
|  | |  | **%** | |
| **Age**** | |  |  | |
| 12-17 | | 8.50 | 12.06 | |
| 18-24 | | 47.67 | 46.60 | |
| 25-29 | | 21.34 | 20.25 | |
| 30-39 | | 19.87 | 18.34 | |
| >=40 | | 2.63 | 2.75 | |
| **Marital Status **** | |  |  | |
| Never Married | | 43.48 | 33.77 | |
| Married/ cohabited | | 51.02 | 62.36 | |
| Divorced / widowed | | 5.51 | 3.87 | |
| **Educational level** | |  |  | |
| Primary or lower | | 8.99 | 9.07 | |
| Secondary/ 9th grade | | 34.18 | 30.82 | |
| High school/ 12th grade | | 39.66 | 42.78 | |
| Greater than high school | | 17.17 | 17.34 | |
| **Occupation **** | |  |  | |
| Unemployed, housewife | | 25.13 | 18.79 | |
| Employed | | 47.1 | 54.77 | |
| Student | | 27.77 | 26.44 | |
| **State of residence **** | |  |  | |
| Mexico City | | 71.36 | 65.26 | |
| State of Mexico | | 23.83 | 26.92 | |
| Other State | | 4.81 | 7.82 | |
| **Number of pregnancies *** | | |  | |
| 1 | | 37.70 | 40.52 | |
| 2-3 | | 44.82 | 41.48 | |
| >=4 | | 17.48 | 18.00 | |
| **Chart Year**** | |  |  | |
| 2007-2009 | | 20.16 | 36.45 | |
| 2010-2012 | | 72.09 | 61.46 | |
| 2013-2015 | | 7.75 | 2.09 | |

Note: ** p<0.01, * p<0.05 for difference between those included and those dropped
